# Generation of VDR Knock-Out Mice via Zygote Injection of CRISPR/Cas9 System

**DOI:** 10.1371/journal.pone.0163551

**Published:** 2016-09-29

**Authors:** Tao Zhang, Yajun Yin, Huan Liu, Weili Du, Chonghua Ren, Ling Wang, Hongzhao Lu, Zhiying Zhang

**Affiliations:** 1 College of Animal Science & Technology, Northwest A&F University, Yangling, Shaanxi, People's Republic of China; 2 School of Bioscience and Engineering, Shaanxi SCI-TECH University, Hanzhong, Shaanxi, People's Republic of China; Qingdao Agricultural University, CHINA

## Abstract

CRISPR/Cas9 system has become a new versatile technology for genome engineering in various species. To achieve targeted modifications at the same site in both human and mice genomes by a CRISPR/Cas9 nuclease, we designed two target sites in conserved regions of vitamin D receptor (VDR) gene, which cover more than 17 kb of chromosome region depending on the species. We first validated the efficacy of single sgRNA mediated gene specific modifications were 36% and 31% in HEK293T cells. Concurrently, targeted of the intervening genomic segments deletions were generated in chromosomes when two sgRNAs worked simultaneously. The large genomic DNA segments up to 23.4 Kb could be precisely deleted in human chromosomes. Subsequently, Cas9 mRNA and sgRNAs targeting VDRT1 and VDRT2 were co-microinjected into one-cell-stage embryos of C57BL/6 mice. Verified by T7E1 assay and DNA sequencing analysis, 12 mice showed VDR targeted disruption and 8 of which were biallelic knock-out, which demonstrated obvious phenotype of hair thinning. Furthermore, expression changes of Vitamin D metabolism genes in VDR^-/-^mice were detected. These results indicated that CRISPR/Cas9 mediated knock-out of VDR diminished its gene function in vivo. The off-target effects of CRISPR/Cas9 in VDR^-/-^ founder mice were analyzed. Our results showed that CRISPR/Cas9 system could be employed to target the same sites in different species, when sgRNAs are designed within conserved regions, and therefore will be critically important and applicable for human disease model.

## Introduction

Vitamin D mediates a variety of biological functions such as calcium homeostasis, calcium reabsorption in the kidney, calcium mobilization in bone, cell differentiation and proliferation to many target tissues[1]. Most, if not all, the biological actions of vitamin D are believed to be exerted through the vitamin D receptor (VDR)-mediated control of target genes [2,3]. Mutations in the *VDR* cause the disease known as hereditary vitamin D resistant rickets (HVDRR) [4]. Through DNA microarray technology, 95 genes were identified that displayed different changes of expression level in *VDR* null mice, of which 28 genes were up-regulated and 67 were down-regulated [5]. Using whole body *VDR*^*-/-*^ mice, intestinal epithelial VDR conditional knockout (VDR(ΔIEC)) mice, and cultured human intestinal epithelial cells, Claudin2 (CLDN2) gene had been demonstrated to be a direct target of the transcription factor VDR [6]. However, the complete profile of *VDR* action is still unknown, and precise targeted editing of *VDR* is critical to understanding the biological functions of *VDR*, which could be the key to development of novel therapeutic modalities for *VDR*-related diseases.

Targeted genomic editing is a powerful technology in revealing gene functions, gene therapy for human diseases, generation of models and breeding animals with desired traits. A novel genome editing platform based on clustered regularly interspaced short palindromic repeats(CRISPR)/CRISPR associated (Cas) protein system provides adaptive immunity against viruses and plasmids in bacteria and archae [7,8]. The type II CRISPR/Cas9 of *Streptococcus pyogenes*is is a relatively simple CRISPR/Cas system, and only involves a single effector enzyme to cleave dsDNA. Given this advantage, it has rapidly been developed into a viable genome editing tool [9]. CRISPR/Cas9 nuclease is distinct from ZFNs and TALNEs, and it mediates genome editing following the rule of targeted DNA recognizing and cleavage by designed short guide RNAs (gRNAs) and endonuclease Cas9, respectively. Feng Zhang developed a plasmid that contained both hspCas9 nuclease and a functional gRNA [10]. Since then, the CRISPR/cas9 nuclease has become a dominant genome editing platform, and has been successfully used to generate target gene modified cells in plants and animals [11–14].

Co-injection of Cas9 mRNA and sgRNA into one-cell stage embryos has been demonstrated to be an efficient approach for the generation of genetically modified animals. In this study, we designed and constructed CRISPR/Cas9 including two target site of VDR gene. Then, the activity of CRISPR/Cas9 was detected in HEK293T cells. Subsequently, through co-injection of one-cell stage embryos of C57BL/6 mice with sgRNAs of two target sites (VDRT1 and VDRT2) and Cas9 mRNA, VDR targeted modifications mice were achieved. Finally, we analyzed the phenotypes, the expression level of Vitamin D metabolism genes and off-target mutations in the VDR knock-out mice. These results displayed that we had efficiency and reliability generation VDR knockout mice via injection of zygotes with Cas9 mRNA and sgRNAs.

## Materials and Methods

### Animals and Ethical statement

C57BL/6 mice were purchased from The Fourth Military Medical University Laboratory Animal Center. The animals were maintained in the Experimental Animal Room of Northwest A&F University. All animal experiments involving the care and use of animals conformed to the U.S. National Institutes of Health guidelines (NIH Pub. No. 85–23, revised 1996) and were approved by the Animal Care and Use Committee of the Northwest A&F University.

### Design of CRISPR/Cas9 plasmid and reporter vector

**Fig 1:**
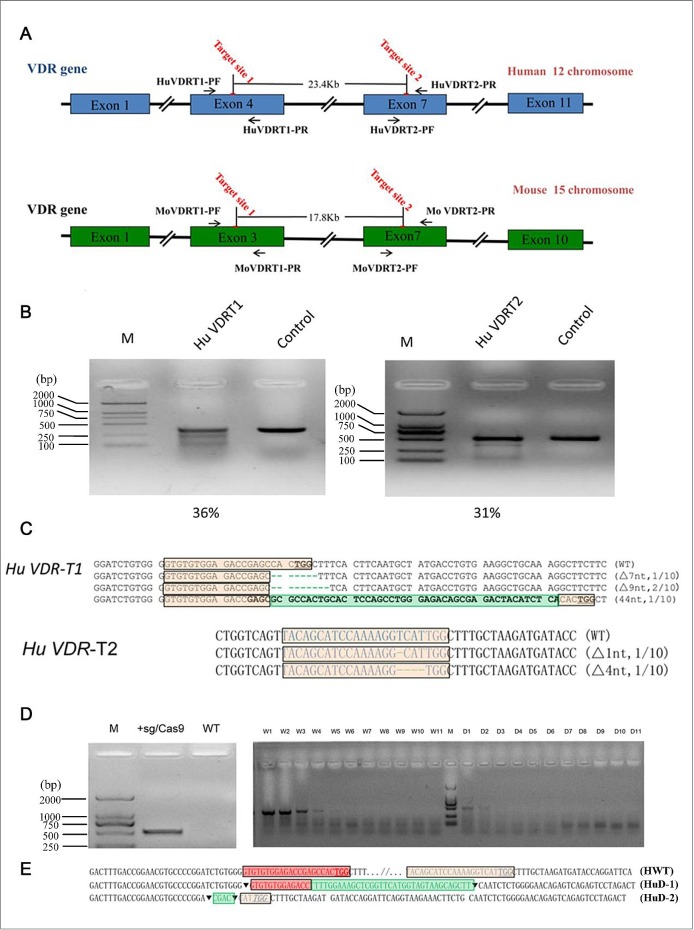
Evaluation of CRISPR/Cas9 mediated cleavage of VDRT1 and VDRT2 in human 293T cells. (A) Schematic diagram of location of two VDR targeting sites in human chromosome. The red lines stand for the locations of two target sites in VDR. The primers for the PCR amplified target sites sequence are indicated by a black arrow. (B) The efficiency of CRISPR/Cas9 mediated cleavage at two target sites in HEK293T cells. (C) CRISPR/Cas9 mediated VDR targeted editing in human 293T cells. Target sequences were highlighted with pink boxes, and PAM sequences were highlighted as underlined. Green boxes stand for insertion sequences (▼, deletion junction; △, deletion; +, insertion). (D) The large fragment deletion was identified by PCR. Left panel, the PCR products of lane 2 (+CRISPR/Cas9) and lane 4 (WT-DNA) were amplified from treated cells and wild type genomic DNA, respectively. Right panel, digital PCR analysis of the large genomic fragment deletions within the VDR. The strong 400bp band in all lanes W1-W11 (on Mark right) is a non-specific amplification product. The 500bp bands from D1-D11 (on Mark right) correspond to the expected size of the PCR product in the event of large fragment deletion of the intervening sequence. (E) Target sequences were highlighted with red boxes and pink boxes, and PAM sequences were highlighted as underlined. Green boxes stand for insertion sequences (▼, deletion junction).

The sgRNA-Cas9 co-expression plasmid pX330-U6-chimeric-dBsaI-CBh-hspCas9 as parent vector was obtained from Addgene (http://www.addgene.org/), which harbors two different sticky ends by BsaI digestion [15]. According to the design principle and program of CRISPR/Cas9 (http://crispr.mit.edu/, Zhang Feng Lab), we designed two target sgRNAs (VDRT1 and VDRT2), which respectively target exon 3 and exon 7 of VDR in mouse genome. Aside from the PAM sequence, the sequences of the two target sites were identical to the sequences of human VDR gene (Fig 1A).

To construct VDR target vectors, the target dsDNA with sticky ends were generated via direct annealing of two oligonucleotides VDRT1F and VDRT1R (S1 Table), in which sticky ends sequences exactly match BsaI ends in Cas9 parent vector. Thus, VDRT1 was cloned into the parent vector between two *BsaI* sites. The plasmid was designated pX330-U6-VDRT1-CBh-hspCas9. Meanwhile, pX330-U6-VDRT2-CBh-hspCas9 as VDRT2 target vector was obtained with the same strategy.

pCAG-puro-NB-T2A-EGFP backbone plasmid was used to construct CRISPR/Cas9 reporter vector, in which puromycin resistant gene (*Puro*^*R*^) was separated by NotI and BamHI enzyme sites flanking with two 200bp direct repeats of *Puro* ^*R*^ (S1 Fig). In order to insert *VDR* target sites into backbone plasmid, two oligonucleotides for each target were designed and synthesized (S2 Table), and target DNA fragments harboring PAM sequence and NotI and BamHI sticky ends were generated by direct annealing. Then *VDR* target fragments were cloned into pCAG-puro-NB-T2A-EGFP between NotI and BamHI sites to achieve two reporter plasmids, designated pCAG-puro-VDRT1-T2A-EGFP and pCAG-puro-VDRT2-T2A-EGFP, respectively.

### CRISPR/Cas9 efficiency test in HEK 293T cells

As the sequences of the two target sites were identical in human and mouse genome, the CRISPR/Cas9 plasmids and corresponding report vectors were transfected into HEK293T cells by NeoFect^TM^ DNA transfection reagent (Neofect biotech, Beijing). According to manufacturer’s instructions, 2 μg Cas9 expression vector and 1 μg report vector were added into each cell culture of 6-well plates. At 24 hour post-transfection, puromycin enrichment was launched to enrich cells containing restored puro^R^ in the reporter vector. After 72 hours for puromycin treatment, cells were maintained in a fresh medium without puromycin for 24 hours, and then the genomic DNA was extracted for PCR, the sequences of primers were listed in S3 Table. Subsequently, T7E1 cleavage assay were performed as previously described [10,16]. Deletion frequencies of large DNA fragment by digital PCR was carried out as previously described [17,18].

### Production of VDR gene knock-out mice

VDR sgRNAs were produced by *in vitro* transcription using the MEGA shortscript kit (Ambion) and purified using the MEGAClear kit (Ambion) according to the manufacturer’s instructions. Using the Cas9 mRNA *in vitro* transcription vector (Addgene No. 44758) as templates, Cas9 mRNA was produced and purified according to description.

Female C57BL/6J mice were injected with pregnant mare serum gonadotropin (PMSG) and human chorionic gonadotropin (hCG) with a 48h interval, and mated with male C57BL/6J mice. Cas9 mRNA (2ng/μL) and sgRNAs (5ng/μL for each sgRNA) targeting VDRT1 and VDRT2 were mixed and injected into the pronuclei of these one-cell-stage embryos according to standard protocols (Gordon and Ruddle 1981). After injection, the zygotes were cultured in M16 Medium for 3h at 37°C, and then the injected one-cell embryos were transferred into pseudopregnant mice.

### T7E1 assay and sequencing of targeted VDR disruption in mice

Total genomic DNA of tails from F0 mice were extracted according to the phenol-chloroform procedure [19]. And then T7E1 assay was performed as previously described to measure targeting efficiencies [10,16]. And DNA sequencing was performed to verify indels after TA cloning into plasmid pMD19T.

### Off-target assay

In order to evaluate off-targeting effects of CRISPR/Cas9 nucleases *in vivo*, we chose six candidate loci for each target site with high potential cleavage in mouse genome via the online program (http://crisppotentialoff-targetr.mit.edu/, Zhang Feng Lab). The selected potential off-target sites were PCR-amplified using genomic DNA from founder animals. The PCR products were then subjected to a T7E1 cleavage assay. The potential off-target sites yielding the typical cleavage bands were further evaluated by TA clone and DNA sequencing. The information on the off-target loci and primer pairs used are listed in S3 and S4 Tables.

### RNA isolation and Real-time PCR

Total RNA was extracted from different tissue of VDR knockout mice and wild type mice via Trizol reagent (TaKaRa, Dalian China). The first-strand cDNA was generated using a reverse transcription kit (TaKaRa) with random primers. Real-time quantitative PCR was performed in triplicate samples using a SYBR green kit (Invitrogen, Thermo Fisher) on the ABI Step one plus system. Mouse *GAPDH* was taken as the reference gene. The 2-ΔΔCT algorithm was employed to estimate the relative expression level of genes. The sequences of primers were listed in S3 Table.

### Immunohistochemistry

5 μm cryosections of small intestine and muscle from VDR^-/-^ and wild type mice were carried out to detect VDR expression. A. Blocking was performed with 1% BSA an d tissue cryosections were further immune stained using the anti-VDR monoclonal antibody D-6 with highly specificity (Santa cruz, sc-13133) overnight. The next day, the sections were washed gently with PBS and incubated with the secondary antibody goat anti rabbit IgG (BOSTER, BA1045). DAPI staining was applied for nuclear staining. The images were captured using a fluorescence microscope (Leica, DMIL/DFC450).

## Results

### Cleavage efficiency of each VDR sgRNA in HEK293T cells

In order to test the cleavage efficiency of CRISPR/Cas9 on target sites in *VDR*, CRISPR/Cas9 expression vectors and their corresponding report plasmids were co-transfected into human 293T cells. After transfection 24 h, Red fluorescence and green fluorescence positive cells were observed (S2 Fig). Meanwhile, puromycin was added into the medium for screening and enrichment of positive clones for 72 h, and cells were harvested to extract total genomic DNA for further analysis. CRISPR/Cas9-induced *VDR* indels were measured using T7 endonuclease I (T7E I), which cleaves heteroduplexes formed by the hybridization of mutant and wild-type *VDR* target sequences or two different mutant sequences. The mutation frequencies of these two *VDR* sites in 293T cells were 36% and 31% by T7E1 assay, respectively (Fig 1B).

To further confirm the mutation frequency induced via CRISPR/Cas9, we cloned the PCR product surrounding the target sites amplified from these enriched cells. Ten clones from each human VDRT1 and VDRT2 sites were randomly picked for direct DNA sequencing. The sequencing results demonstrated that random indels were detected in 4 colonies of VDRT1 and 2 colonies of VDRT2, respectively. Deletion of 9 nt was observed in two independent colonies of VDRT1, though 7 nt deletion was verified in only 1 colony (Fig 1C). Additionally, 44 nt insertion was also found in VDRT1. By comparison, only deletions of 1 nt and 4 nt were detected in VDRT2 site (Fig 1C).

Moreover, we contemplated whether the large chromosome segment between VDRT1 and VDRT2 sites could be deleted by co-transfecting plasmids expressing two sgRNAs in addition to Cas9. If a large DNA fragment had been deleted from the chromosome, a 500 bp DNA fragment should be amplified. Otherwise, no product could be amplified when wild-type chromosomes were used. We detected roughly 500 bp PCR products using primers VDRT1PF and VDRT2PF from Cas9 treated cell genomic DNA, and no products were obtained using wild-type cell genomes as templates (Fig 1D). The PCR products were characterized further by Sanger sequencing, which indicated that large fragment could be deleted in genome using CRISPR/Cas9 (Fig 1E). All these data demonstrated that the designed sgRNAs work efficiently with Cas9 on targeted VDR in HEK 293T cells.

### Generation of VDR knock-out mice

To generate VDR knock-out mice, the Cas9 mRNA and sgRNA mixture targeting VDRT1 and VDRT2 was injected into the cytoplasm of 241 one-cell-stage embryos obtained from C57BL/6J mice. After injection, zygotes were cultured in M16 medium, no morphological abnormalities were observed in 203 of 241 one-cell embryos (84.2%). And then, the normal zygotes were transferred into 8 pseudopregnant mothers. After full-term gestation, which lasted around 21 days, and 33 neonates were obtained from surrogates female mice (Table 1).

**Table 1:** Summary of production of VDR gene modified mice via CRISPR/Cas9.

Strain of embryo	Concentration of RNA(ng/μL)	Cas9-sgRNA injected eggs (one-cell-stage)	Transferred embryos	Surrogate mice	Newborn mice	VDRT1 modified mice	VDRT2 modified mice	VDRT1 modified mice (male)	VDRT1 modified mice (Female)
C57Bl/6J	50(VDRT1)+ 50(VDRT2)+ 100(Cas9)	241	203	8	33	12	0	4^-/-^,10^-/-^, 17^-/+^,26^-/-^, 30^-/+^	7^-/-^, 9^-/-^, 3^-/+^, 14^-/-^, 22^-/+^,29^-/-^, 32^-/-^

**Fig 2:**
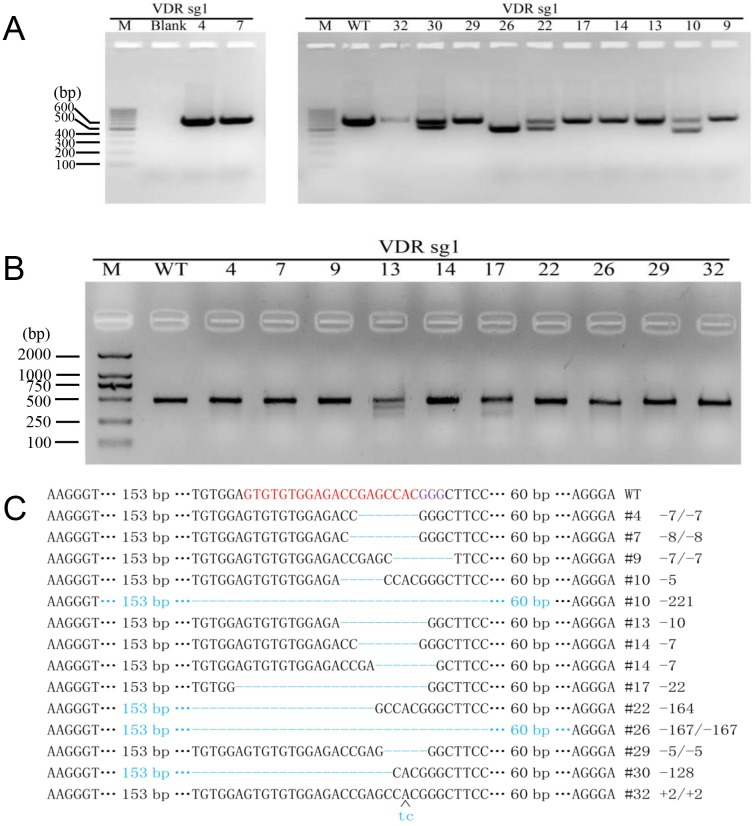
Detection of CRISPR/Cas9 mediated modifications of VDR in mice. (A) PCR products of the targeted region of VDRT1 from founder mice. (B) Detection of cleavage of VDRT1 by T7E1 assay. (C) Sequencing results of re-edited VDRT1 loci detected in founder mice.

The 33 founders were genotyped by PCR followed by DNA sequencing analysis. The PCR and DNA sequencing revealed that genomic modification occurred at VDRT1 in 12 founder mice (33.3%), as expected, different types of indels were observed, but no mutation was detected at VDRT2 in all founder mice (Fig 2A and 2B). Impressively, the sequencing results indicated that VDRT1 mutation was detected at the pair of homologous chromosomes in 6 mice (50%), such as #4, #7, #9, #14, #29 and #32 (Fig 2C). The additional bands were observed by PCR amplification of the target region in 4 mice(33.3%), such as #10, #22, #26, #30 (Fig 2A and 2C), these appearances showed that more than 100 bp DNA fragment deletions in the chromosome. To further confirm their genotypes, the target region was performed by T7E1 assay, TA-cloning and DNA sequencing. The screen determined that 8 biallelic VDR gene knockout mice (66.7%) were generated via zygote injection of CRISPR/Cas9 system (Fig 2C).

### Phenotype and expression levels of Vitamin D metabolism genes in founded mice

**Fig 3:**
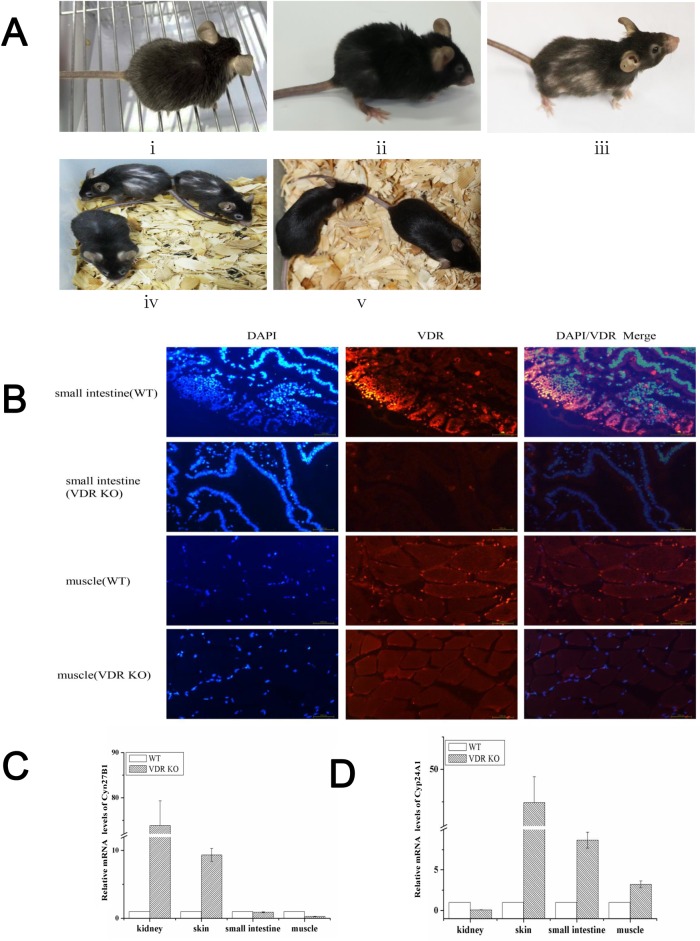
Expression levels of VDR, Cyp27B1 and Cyp24A1 in different tissues from founder mice. (A) Phenotypes of VDR knock-out mice with different ages. (i,ii,iii) VDR^-/-^ mouse #4 at two, three and four months old, respectively; (iv) VDR^-/-^ mice #26,#9 and VDR^-/+^ #17 mouse at four months old; (v) wild type mice at four months old. (B) immunofluorescence histochemistry of VDR in mouse small intestine and muscle. Up panel, D-6/DAPI colocalization in the sections of small intestine. Down panel, D-6/DAPI colocalization in the sections of muscle. (C) The differential expression of Cyp27B1 of kidney, skin, small intestine and muscle by qPCR between VDR knockout mice and WT mice. (D) The differential expression of Cyp24A1 in mouse kidney, skin, small intestine and muscle.

The 33 mice had no obvious differences when when they bron. With increasing in age, the phenotype of hair and bone had shown significant differences between wild type and the VDR knock-out mice. The hair of VDR^-/-^ mice lost its luster at 2 months of age. Loss of hair, bone thinning and atrophy of skin began from 3–4 months of age, then the VDR^-/-^ mice became worse with increasing age and featured like an elderly mice (Fig 3A). Another interesting effect has been the loss of fertility in these VDR knock-out mice. In order to further clarify the effect of VDR targeting, we analyzed the expression of VDR in muscle and small intestine via immunofluorescence histochemistry. The result showed that VDR was not detectable in VDR^-/-^ mice (Fig 3B). Meanwhile, we explored gene-expression differences of 25-hydroxyvitamin D3 1alpha-hydroxylase (CYP27B1) and 24-hydroxylase (Cyp24A1) in kidney, skin, small intestine and muscle between wild type and VDR^-/-^ mice (Fig 3C and 3D). Consistent with previous findings [5,20], the two key enzymes that were involved in vitamin D metabolism, CYP27B1 and Cyp24A1,were changed dramatically in opposite directions in VDR^-/-^ mice kidney. However, the expression levels of CYP27B1and CYP24A1 were all significantly increased in skin after VDR knock-out. And in small intestine and muscle, we found that expressions of CYP27B1 were decreased and CYP24A1 were increased in KO mice, in which the trend was exactly opposite to kidney. All which largely accounted for the difference of function and patterns of expression of the two genes in different tissues and organs. Another thing worth noting is that these founded VDR knockout mice were sterile. So we speculated that VDR might link to fertility. On all accounts, these results indicated that microinjection with sgRNA and Cas9 mRNA can induce complete VDR-/- phenotype in mice.

### Off-targeting effect of CRISPR/Cas9 in mice

**Fig 4:**
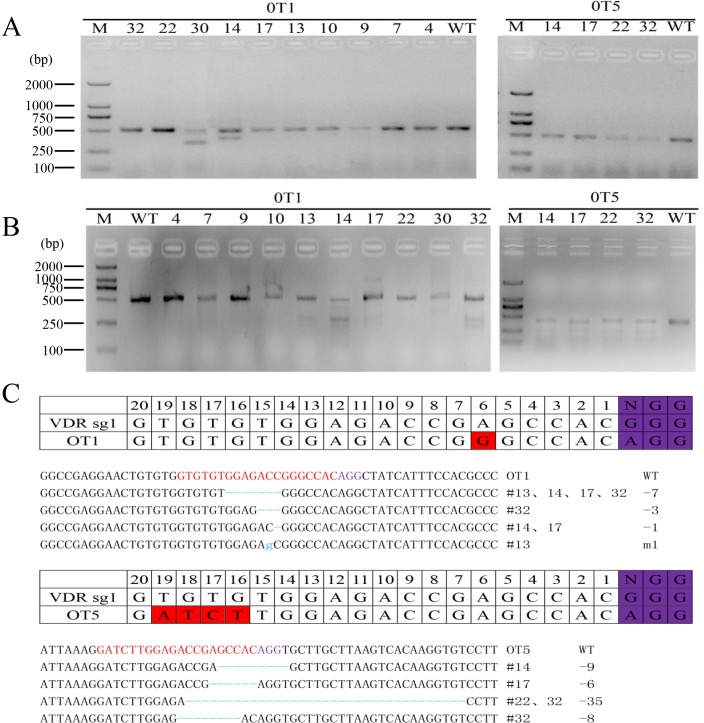
Detection of the CRISPR/Cas9 mediated off-target cleavages *in vivo*. (A) PCR products of the potential off-target sites from founder mice. OT1 and OT5 were selected and amplified from genomic DNA of VDR knockout mice. (B) Detection of off-target cleavage by VDRT1 sgRNA at OT1 and OT5 by T7E1 assay. (C) Sequencing results of PCR products.

Some studies have indicated that CRISPR/Cas9 presents relative high risk of off-target mutagenesis occurred in human cells, mouse and other organisms. To test whether off-target mutations occurred in these founder mice, 6 potential off-target sites were selected for detection (S4 Table). The PCR products at 6 potential off-target sites were subjected to the T7E1 cleavage assay from 12 founder mice genomic DNA. We found that two sites OT1 and OT5 showed off-target modifications in mice genome (Fig 4A and 4B). To further verify the off-target cleavage events, PCR products from disrupted animals were subjected to TA clone. DNA sequencing results displayed that #10, #13, #22, #26, #30 founders had off-target modification at OT1 and #4, #7, #9, #14, #29, #32 founders had off-target modification at OT5 (Fig 4C).

## Discussion

VDR specific sites editing cell lines or model animals could be widely used in VDR biologic function exploring, VDR relative diseases research and novel medication development. CRISPR/Cas9 nucleases are powerful tools for precise genomic editing in various cells and species. We presented the CRISPR/Cas9 system effectively modified conserved sequences in human and mouse VDR with relatively high efficiency. The cleavage activity of the pX330-U6-chimeric-dBsaI-CBh-hspCas9 vector was already confirmed [21], hereby, we engineered pX330-U6-VDRT1-CBh-hspCas9 and pX330-U6-VDRT2-CBh-hspCas9 and successfully modified VDR in human cells. To achieve much more nuclease-targeted cells, a dual-gene report system was employed to screen and enrich genetically modified cells.

Except PAM sequences, these two target sites in human genome are much different. By comparison, it is easy to understand why the efficiency of CRISPR/Cas9 varied greatly between VDRT1 and VDRT2 sites in HEK293T cells. CRISPR/Cas9 induced VDRT1 mutation efficiency via NHEJ in 293T cell was 36%, which was higher than the mutation rate 31% in VDRT2. We speculated that different positions of *VDR* target sites in human chromosome might affect CRISPR/Cas9 cleavage activities. Thus, these results suggest that chromosomal structure might play an important role in regulation of CRISPR/Cas9 activity.

Taken the advantage of multiple sites target editing, CRISPR/Cas9 systems greatly facilitate large DNA fragment deletion in chromosomes [22]. Hereby, CRISPR/Cas9 nucleases targeting VDRT1 and VDRT2 were introduced into HEK293T cells together to achieve large fragments deletion in chromosome. And fragments of 23.4Kb between VDRT1 and VDRT2 were deleted in human chromosomes. The efficiency of 23.4 Kb DNA segment deletion in 293T cells was up high to 10% (Fig 1D), which was much higher than deletion of chromosomes in *Caenorhabditis elegans* [23] and rice [24]. By comparison, another report demonstrated that the efficiency of 65 Kb large DNA fragment deletion was as high as 11.8% in mouse embryonic stem cells via microinjection of CRISPR/Cas9 expression plasmids. According to sequencing results, the breakpoint junctions indicated that CRISPR/Cas9 mediated DSBs were repaired by NHEJ mechanism, a noticeable disparity compared with the NHEJ repairing DSBs induced by ZFN and TALENs [25]. It is likely that Cas9 has the characteristic of cleavage DNA between the third and fourth base pairs in the upstream of the PAM and generates blunt ends [9], and the ligation of two blunt DNA ends may not require a micro-homology alignment process.

Kato et al generated VDR-deficient mice by homologous recombination in mouse embryonic stem (ES) cells [26], but the conventional gene-targeting methods is costly and time consuming to produce gene knockout mice, and difficult to obtain double-mutant mice. The current study provides evidence that CRISPR/Cas9 system is a precise and versatile tool for genome editing in various species. Wang et al first generated Tet1 and Tet2 genes mutation mice by zygote microinjection of sgRNA and Cas9 mRNA[27]. When they obtained 28 pups from 144 embryos transferred into foster mothers (21% live-birth rate), and 22 (78.6%)mice carrying targeted mutations at all four alleles of the Tet1 and Tet2 genes, 4(2.6%) mice carrying three alleles targeted mutations. In order to generate VDR targeted mouse quickly and efficiently, we use the CRISPR/Cas9 system to targeted VDRT1 and VDRT2 in C57BL/6J mice by pronuclear microinjection of Cas9 mRNA and sgRNAs. Here, we produced 33 pups from surrogate mothers, and 12 pups carry VDRT1 mutations, and 8 (66.7%) mice carrying targeted mutations at two alleles of VDRT1, but no VDRT2 targeted in all pups. We thought that the reason largely accounted for the sequence of sgRNA, sgRNA of VDRT1 mediated off-target cleavages were detectable in two potential off-target sites. It could reduce the work efficiency of CRISPR/Cas9 on the target site. Some research display that sgRNA expression through the commonly used U6 promoter requires a guanosine nucleotide to initiate transcription, thus constraining genomic-targeting sites to GN19NGG. In our experiment, the 5’ end of VDRT2 sgRNA was not a G, which may affect the efficiency and integrality of the sgRNA transcription in vitro. Thus VDRT2 knock-out mice were not achieved after co-injection. Through the efficiency in this study was a little lower than Wang et al, but our results reconfirmed that pronuclear microinjection of Cas9 mRNA and sgRNA provided highly efficient induction of NHEJ-mediated mutations. And we should carefully choose and evaluate sgRNA to increase cleavage efficiency and avoid the off-target effect in genome editing research.

Previous studies reported that high off-target effects were detected in the whole genome, especially in cultured cell lines. Recently, several groups revealed that off-target cleavage of CRISPR/Cas9 occurred at some genomic sites that differ with up to five nucleotides from the target sites [28]. The high off-target effects of the CRISPR/ Cas9 system is the concern on the application of CRISPR/Cas9 system *in vivo* (Fu et al. 2013). In the present study, 6 potential off-target loci were selected to detect off-target efficiency in founder pups, and two sites OT1 and OT5 showed off-target modifications in #13, #14, #17, #22, #30, #32 mice. To further analyze the off-target sequences, the two sites with obvious off target effect all poss NGG PAM motifs. For protospacer, the point mutations within the ‘seed sequence’ (the 8 to 10 protospacer nucleotides immediately adjacent to the PAM) could abolish CRISPR targeting activity. The distal (from the PAM) positions of the protospacer (12 to 20) could tolerate most mutations [9,29,30]. However, only one mismatch of OT1 at 6nt to PAM was in the seed sequence, which displayed a relatively high off-target effect in VDR knock-out mice. In OT5, we found that t the mismatch of 2–5 consecutive four nucleotides located in the 5’ end of the 20 nt sgRNA also occurred off-target cleavage in mouse genome. Scientists have developed various strategies to avoid CRISPR/Cas9 off-target effects. Cas9 and sgRNAs were modified or engineered to reduce off-target effects. Many different softwares have been developed for target selection and potential off-target evaluation [31–34]. Off-target DNA cleavages can also be prevented by replacing nucleases with nickases[35,36]. Cas9 can be converted to a nickase by mutation of some key amino acid residues at active domains [37]. In addition, Mashiko et al showed that very few off-target mutations occurred by pronuclear microinjection of px330 plasmid DNA in mice [38]. These data suggested some basic information in off-target research of CRISPR/Cas9 and choosing optimal target sites.

One of the main goals of targeted gene modification is to achieve an ideal model for revealing gene functions. Earlier research had reported the differences of gene expression profile in the kidney between Vitamin D receptor knockout mice and wild type[5]. They founded that the expression level of CYP27B1 increased, and the expression level Cyp24A1 reduced. Our results were consistent with the report. In addition, we found that the expression profiles of Cyp27B1 and Cyp24A1 indicated the different changing trends in skin, small intestine and muscle from VDR-/- mice. These findings may have important practical ideas for Vitamin D metabolism and VDR function in different tissues. In these VDR-/- mice, VDR expression level was undetectable in small intestine and muscle, and some phenotype of VDR-/- mice were in consistent with VDR deficiency, such as loss of hair, bone thinning, atrophy of skin, sterility, and aging. Vitamin D receptor (VDR) is a nuclear transcription factor responsible for the biological activity of vitamin D, and present in a diverse range of tissues. VDR has the ability to regulate gene transcription and stimulate intra-cellular signaling pathways, such as cWnt, Hedgehog, mTOR and TGF-β. In our study, these phenomena indicated that CRISPR/Cas9 mediated mutations of VDR diminished its function in vivo, which could provide an alternative model for VDR function research in further research.

In summary, high-efficiency production of VDR knockout mice was achieved by microinjection of C57BL/6 zygotes with Cas9 mRNA and sgRNA. Although there are still several problems to solve, such as reducing off-target effect and optimizing CRISPR/Cas9 cleavage efficiency, the present study suggested that CRISPR-Cas9 system could be used to efficiently produce VDR knock out mouse models in which to investigate gene functions.

## Supporting Information

S1 Fig Schematic diagram of reporter vector characteristics. DsRed gene was a reporter gene to test the transfection efficiency of CRISPR/Cas9 system. During the introduction of CRISPR/Cas9, the disrupted puromycin resistance gene (Puro^R^) was repaired by single strand annealing (SSA), resulting in restored Puro^R^ and eGFP.
(TIF)

S2 Fig Validating activity of CRISPR/Cas9 in HEK293T cells. HEK293T cells were transfected with expression vectors and their corresponding reporter vectors to validate nuclease activities. Red fluorescence indicated vectors delivery into cells successfully. And green fluorescence implied designed CRISPR/Cas9 nuclease cut target sites in reporter vectors.
(TIF)

S1 Table Oligonucleotides of sgRNA for CRISPR/Cas9 expression plasmids construction. * Restriction enzyme recognition sequences are in lower-case.
(DOCX)

S2 Table Oligonucleotides of target site for report vector construction. * Restriction enzyme recognition sequences are in lower-case. Underlined nucleotides are nucleotides in the protospacer adjacent motif (PAM) following the 20-ntsgRNA targeting sequence.
(DOCX)

S3 Table PCR primers for target and off target sequences amplification. (DOCX)

S4 Table The sequences of potential off-target sites in mouse genome. *PAM is indicated in underline. Mismatches Nucleotide between the target sequence and the potential off-target sequences are in lower-case.
(DOCX)
