# Supplementary figures and images for: Generation of VDR Knock-Out Mice via Zygote Injection of CRISPR/Cas9 System
Source: PLoS One. 2016 Sep 29;11(9):e0163551. doi: 10.1371/journal.pone.0163551 (PMC5042489; doi:10.1371/journal.pone.0163551)

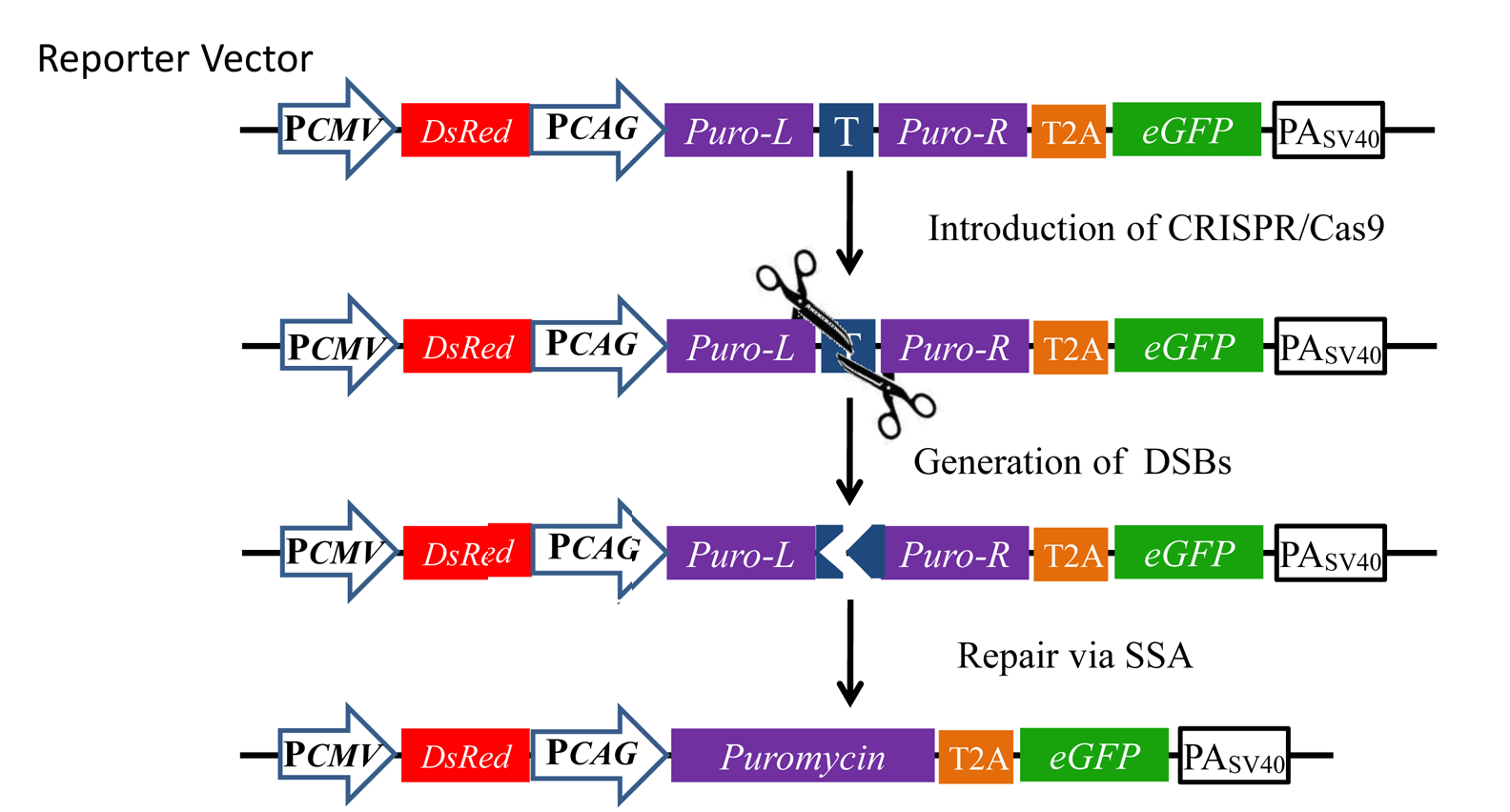

Supplement: S1 Fig — DsRed gene was a reporter gene to test the transfection efficiency of CRISPR/Cas9 system. During the introduction of CRISPR/Cas9, the disrupted puromycin resistance gene (PuroR) was repaired by single strand annealing (SSA), resulting in restored PuroR and eGFP. (TIF) [file pone.0163551.s001.tif]

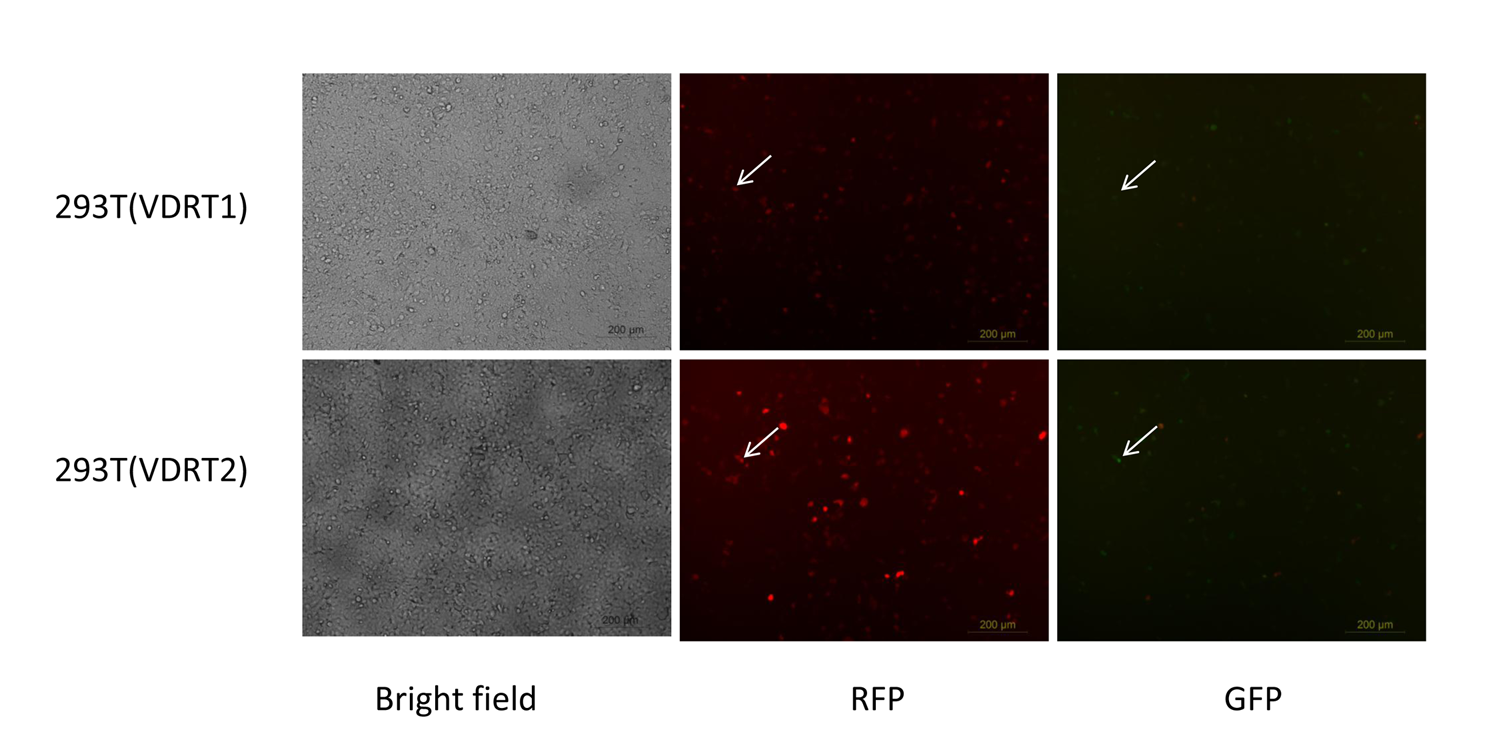

Supplement: S2 Fig — HEK293T cells were transfected with expression vectors and their corresponding reporter vectors to validate nuclease activities. Red fluorescence indicated vectors delivery into cells successfully. And green fluorescence implied designed CRISPR/Cas9 nuclease cut target sites in reporter vectors. (TIF) [file pone.0163551.s002.tif]
