# Supplementary material for: Generation of VDR Knock-Out Mice via Zygote Injection of CRISPR/Cas9 System
Source: PLoS One. 2016 Sep 29;11(9):e0163551. doi: 10.1371/journal.pone.0163551 (PMC5042489; doi:10.1371/journal.pone.0163551)
Supplement: S1 Table — * Restriction enzyme recognition sequences are in lower-case. (DOCX) [file pone.0163551.s003.docx]

**S1 Table**

| Name | Sequences* | Note |
| --- | --- | --- |
| VDRT1F : | *cacc* GTGTGTGGAGACCGAGCCAC | For sgRNA/Cas9-VDRT1 |
| VDRT1R : | *aaac* GTGGCTCGGTCTCCACACAC | For sgRNA/Cas9-VDRT1 |
| VDRT2F : | *cacc* TACAGCATCCAAAAGGTCAT | For sgRNA/Cas9-VDRT2 |
| VDRT2R : | *Aaac*ATGACCTTTTGGATGCTGTA | For sgRNA/Cas9-VDRT2 |
