# Supplementary material for: Generation of VDR Knock-Out Mice via Zygote Injection of CRISPR/Cas9 System
Source: PLoS One. 2016 Sep 29;11(9):e0163551. doi: 10.1371/journal.pone.0163551 (PMC5042489; doi:10.1371/journal.pone.0163551)
Supplement: S2 Table — * Restriction enzyme recognition sequences are in lower-case. Underlined nucleotides are nucleotides in the protospacer adjacent motif (PAM) following the 20-ntsgRNA targeting sequence. (DOCX) [file pone.0163551.s004.docx]

**S2 Table**

| Name | Sequences* | Note |
| --- | --- | --- |
| VDR-ReT1F | ggccgcGTGTGTGGAGACCGAGCCACTGG g | For VDRT1 reporter vector |
| VDR-ReT1R | gatccCCAGTGGCTCGGTCTCCACACAC*gc* |  |
| VDR-ReT2F: | ggccgcTACAGCATCCAAAAGGTCATTGG g | For VDRT2 reporter vector |
| VDR-ReT2R: | gatccCCAATGACCTTTTGGATGCTGTAgc |  |
