# Supplementary material for: Generation of VDR Knock-Out Mice via Zygote Injection of CRISPR/Cas9 System
Source: PLoS One. 2016 Sep 29;11(9):e0163551. doi: 10.1371/journal.pone.0163551 (PMC5042489; doi:10.1371/journal.pone.0163551)
Supplement: S3 Table — (DOCX) [file pone.0163551.s005.docx]

**S3 Table**

| Category | Name | Sequence | Note |
| --- | --- | --- | --- |
| For designated targets | HuVDRT1-PF | TGCTTGCTGTTCTTACAGGGAT | For human VDRT1 target sequence |
|  | HuVDRT1-PR | CAGAGGAACATCTGGAGCTGAG |  |
|  | HuVDRT2-PF | CAGACATGATGGACTCGTCCAG | For human VDRT2target sequence |
|  | HuVDRT2-PR | GAGCGAGAATCTGTCTGGAAAA |  |
|  | MoVDRT1PF | CGGTGGCTATGCTGAAGGTG | For mouse VDRT1 target sequence |
|  | MoVDRT1PR | GACACGGTGGGACTGAGAAG |  |
|  | MoVDRT2-PF | CTGGGTGTCCTTAAATAGCTCTC | For mouse VDRT2 target sequence |
|  | MoVDRT2-PR | AAGATCTTAGGTGGCCATGAGAC |  |
| For potential off-site targets | MoVDRT1OT1-PF | ACATTTTTGCTTTTAGTGTCCGC | For VDRT1 sgRNA in mouse genome |
|  | MoVDRT1OT1-PR | TGTTCTTGACTCTTAGGCTCTG |  |
|  | MoVDRT1OT2-PF | TGGTGATGTTGTAACCGGCTTT | For VDRT1 sgRNA in mouse genome |
|  | MoVDRT1OT2-PR | TCCGTAGAAGGAGCTGGAAGTG |  |
|  | MoVDRT1OT3-PF | TCAGCCTGCTTAGTGTTATG | For VDRT1 sgRNA in mouse genome |
|  | MoVDRT1OT3-PR | TCTGCAAGGACAAGATGATG |  |
|  | MoVDRT1OT4-PF | GGTTAGCGTGGCACATTAC | For VDRT1 sgRNA in mouse genome |
|  | MoVDRT1OT4-PR | TGGTGTAGTCCTGGCTGTC |  |
|  | MoVDRT1OT5-PF | CACATCCACCTCTACCCT | For VDRT1 sgRNA in mouse genome |
|  | MoVDRT1OT5-PR | CCTTATCCTAGACCTACCG |  |
|  | MoVDRT1OT5-PF | TTCCTTGCTTTGGGTGGTG | For VDRT1 sgRNA in mouse genome |
|  | MoVDRT1OT5-PR | TAGTCAGAGCAGGAGCTAAGAGT |  |
|  | M13R | AGCGGATAACAATTTCACACAGGA | For TA clone sequence |
| For qPCR | CYP27B1F | AACTCTTCTGCAAAGGGGGGC | For mouse CYP27B1 |
|  | CYP27B1R | CTGTGCGAAGTGTCCCAAAGC |  |
|  | CYP24A1F | AGCTGCACAAGCGCCTCAAC | For mouse CYP24A1 |
|  | CYP24A1R | CACAAAGGAAATCCGCACCAG |  |
