# Supplementary material for: Generation of VDR Knock-Out Mice via Zygote Injection of CRISPR/Cas9 System
Source: PLoS One. 2016 Sep 29;11(9):e0163551. doi: 10.1371/journal.pone.0163551 (PMC5042489; doi:10.1371/journal.pone.0163551)
Supplement: S4 Table — *PAM is indicated in underline. Mismatches Nucleotide between the target sequence and the potential off-target sequences are in lower-case. (DOCX) [file pone.0163551.s006.docx]

**S4 Table**

| Name | Sequence* | Note |
| --- | --- | --- |
| MoVDRT1-OT1 | GTGTGTGGAGACCGgGCCACAGG | The off-sites target1 for VDRT1 sgRNA in mouse genome |
| MoVDRT1-OT2 | GgGTGgGGcGACCGAGCCACAGG | The off-sites target 2 for VDRT1 sgRNA in mouse genome |
| MoVDRT1-OT3 | GatTtTaGAGACCGAGCCACTGG | The off-sites target 3 for VDRT1 sgRNA in mouse genome |
| MoVDRT1-OT4 | aTGTcTGGgGACCGAGCCACTGG | The off-sites target 4 for VDRT1 sgRNA in mouse genome |
| MoVDRT1-OT5 | GatctTGGAGACCGAGCCACAGG | The off-sites target 5 for VDRT1 sgRNA in mouse genome |
| MoVDRT1-OT6 | GTGccTGGAGACtGAGCCACAGG | The off-sites target 6 for VDRT1 sgRNA in mouse genome |
